# Effects of Processing Technology on Protein Separation, Quality, and Functional Characteristics

**DOI:** 10.3390/foods12203841

**Published:** 2023-10-20

**Authors:** Zhongjiang Wang, Fuwei Sun

**Affiliations:** College of Food Science, Northeast Agricultural University, Harbin 150030, China; sfwname@163.com

## 1. Introduction

Proteins provide the material foundation of all life activities and play an important role in the physiological and biochemical metabolism of the human body. Based on their origins, proteins can be categorized into animal proteins and plant proteins. In recent years, there have been growing concerns about the high cost and negative impact on the environment associated with animal proteins. Plant proteins are of interest to the food science fields due to their biocompatibility, biodegradability, biosafety, and sustainability. According to estimates, the plant protein sector is projected to account for approximately 7.7% of the worldwide protein market within the next ten years. This industry is expected to grow from USD 29.4 billion in 2023 to USD 162 billion by 2033 [1]. By 2050, it is anticipated that the demand for plant protein will rise by USD 9.8 billion due to the growth in the global population. It has been shown that the consumption of plant proteins lowers the incidence of many metabolic problems, including diabetes, cancer, and heart disease [2]. Moreover, plant proteins exhibit significant functional characteristics such as solubility, gelling properties, and emulsifying properties, along with biological activities such as antioxidant properties and flavor adsorption characteristics. Hence, plant proteins could fulfill the sensory and nutritional demands of consumers, as well as the developmental necessities of various food items.

Different types of plant proteins can be categorized based on their source, including proteins derived from nuts (such as walnuts, peanuts, almonds, and cashews), legumes (including soybeans, peas, and chickpeas), seeds (such as sesame, sunflower, and flaxseed), and cereals (such as rice, wheat, and maize). Typically, plant proteins must be extracted from plant cells in order to enhance their commercial value. Presently, the crude protein extracts acquired through traditional extraction techniques, such as organic and inorganic solvents, are no longer capable of satisfying the market requirements. The extraction of plant proteins can be rendered more sustainable and effective through the use of novel technologies, such as ultrasound, high hydrostatic pressure, microwave-assisted extraction, and ion-exchange chromatography. Advanced techniques for isolation and refinement have the potential to generate proteins of exceptional purity, facilitating comprehensive investigations into their spatial architectures and active regions and the correlation between their structure and functionality.

The functional properties of plant proteins, such as solubility, emulsification, and film formation, are inferior, thus restricting their application in food matrices. Appropriate processing and modification can enhance the functionality and physicochemical properties of plant proteins. Currently, commonly used modification methods include physical modification (thermal treatment, ultrasound treatment, and high-pressure homogenization), chemical modification (glycosylation, acylation, succinylation, deamidation, and phosphorylation), and biological modification (fermentation and enzyme catalysis). Although traditional processing techniques have also improved the functionality of plant proteins, there are still obstacles to obtaining high-quality functional plant proteins. Nevertheless, the utilization of emerging processing techniques has garnered significant interest in the domains of both protein extraction and protein modification, including high hydrostatic pressure, atmospheric cold plasma, high-moisture extrusion, ohmic heating, pulsed ultraviolet light, and interaction with small molecules [2]. These processing technologies show promise in boosting the advancement of the plant protein industry.

## 2. Limitations of the Current Processing Technologies for Plant Proteins

At present, traditional techniques for processing plant proteins mostly operate by adjusting the environment conditions of proteins. For example, through the changing of the pH value, ion strength, temperature, and other environmental conditions of protein solutions, the resource utilization of plant proteins can be enhanced to a large degree, even though these are also the condition parameters used in the protein extraction process. However, processing methods under polar conditions can not only easily accelerate the denaturation of plant proteins, but also destroy the function and nutrition of food ingredients while affecting the natural taste and flavor of food and generating toxic by-products and decreasing the bioavailability of essential amino acids [3]. Long-term extreme heating conditions can cause irreversible changes in the protein structure, leading to an excessive denaturation and decrease in its functionality. Polar pH conditions (a higher or lower pH) can also cause proteins to change their natural conformation, forming aggregates with tighter binding and larger particle sizes. The commonly used alkaline extraction technology has also been proven to consume a large amount of water resources, accompanied by a significant loss in heat-sensitive micronutrients that are rich in vitamins and minerals. In addition, some amino acid residues undergo transformation, forming toxic compounds [4]. Therefore, although traditional protein-processing techniques can effectively achieve processing objectives, they could have negative effects on environmental sustainability and the protein’s nutritional and techno-functional properties. To address their limitations, scientists have devised multiple green processing technologies. Utilizing innovative technologies in the processing of plant proteins, as opposed to conventional methods, has the potential to improve the resource utilization of plant proteins while preserving their techno-functional and quality characteristics.

## 3. The Impact of Emerging Processing Technologies on Plant Protein Separation and Functional Modifications

In the past, pure plant proteins have been created for use in the food industry and for biomedical applications. Nevertheless, plant proteins commonly occur naturally as blends, meaning that we need to extract and refine them while preserving their structures and functionalities. Numerous techniques exist for separating and purifying proteins, and these rely on variations in the protein shape, molecular size, and charge. The major drawbacks to these methods are the high cost of large-scale production, the complex procedures, and environmental pollution, despite their ability to generate small amounts of highly purified proteins. Novel extraction techniques could further provide higher purity, lower solvent usage, a lower energy consumption, and reduced effluent. For instance, the use of enzymes in the extraction process can retrieve proteins of a superior quality from plant matrices, enhancing their functional properties. The microjets and turbulence effects generated via ultrasonic-assisted extraction can diminish the degradation or denaturation of proteins, thus leading to the extraction of high-quality proteins [5]. Pulsed-electric-field-assisted extraction is a novel technique that offers numerous benefits, including rapid processing, the absence of chemicals, reduced energy usage, increased yield, and environmental sustainability. Although these new technologies are considered safe and environmentally friendly, they have not yet been fully utilized to extract plant proteins. Therefore, we should vigorously promote these novel green extraction technologies.

The dynamic changes to plant proteins through food processing and production have a great effect on proteins’ functional properties. Some plant proteins have poor functional properties (solubility, emulsifying, gelation, and rheology properties), which severely limit their application in the food industry. Li et al. [6] found that appropriate heat treatment of the Phaseolus vulgaris L. protein could loosen and disorder the protein structure, thereby promoting its binding ability at the oil–water interface and improving its functional characteristics. Traditional processing technologies may cause a decline in the sensory quality of proteins and adverse health effects. Therefore, some emerging technologies for plant protein processing will soon replace traditional processing technologies. For example, atmospheric cold plasma, high-moisture extrusion, and ohmic heating are recently developed technologies that suit the processing of plant proteins [7,8]. Although there are some studies showing that emerging technologies for plant protein processing could improve the functional, structural, nutritional, and rheological properties of proteins, we still need to explore the influential mechanism.

## 4. Prospects for Emerging Technologies for Plant Protein Processing

Due to the continuously growing population, the food sector has begun searching for more affordable, nutritious, and premium protein sources in order to fulfill the dietary requirements of individuals. The study of high-quality plant proteins with excellent functional properties has become a popular research field. Exploring emerging technologies for plant protein processing is a pressing issue. Studies have reported that chemical modifications of plant proteins could potentially increase the levels of toxicity and the risk of the development of food allergies. The utilization of new methods of processing plant proteins offers benefits in relation to enhanced safety, reduced allergenicity, and minimized adverse effects on the physicochemical and functional properties of the proteins [8]. However, most of these methods have not yet been widely used in industry and are only being applied on a laboratory scale. Hence, it is essential to conduct research and development work to optimize these emerging technologies in order to increase their application in plant protein processing. An example comprises the renovation of traditional protein-processing equipment and the optimization of the equipment parameters to make it more suitable for industrial production. It is important to highlight that the efficient utilization of these advanced techniques for plant proteins will also require a thorough comprehension of the correlation between the structural variety of a plant protein and equipment parameters, thus helping to precisely design equipment for plant protein processing to meet industrial needs. In addition, more comprehensive studies are required to further understand the economic viability and cost-effectiveness of the scale-up processes for these emerging technologies for plant protein processing, which will be conducive to rapid development in the plant protein sector.

This Special Issue aims to publish quality articles on a wide range of aspects of technologies for plant protein processing, including their role in protein separation, quality, and functional characteristics.
